# Prediction of Clinical Outcomes with Explainable Artificial Intelligence in Patients with Chronic Lymphocytic Leukemia

**DOI:** 10.3390/curroncol30020148

**Published:** 2023-02-04

**Authors:** Joerg Hoffmann, Semil Eminovic, Christian Wilhelm, Stefan W. Krause, Andreas Neubauer, Michael C. Thrun, Alfred Ultsch, Cornelia Brendel

**Affiliations:** 1 Department of Hematology, Oncology and Immunology, Philipps University Marburg, University Hospital Giessen and Marburg, Baldingerstrasse, 35043 Marburg, Germany; 2 Department of Medicine 5, Universitätsklinikum Erlangen, Maximiliansplatz 2, 91054 Erlangen, Germany; 3 Databionics, Mathematics and Computer Science, Philipps University Marburg, Hans-Meerwein-Strasse 6, 35032 Marburg, Germany

**Keywords:** chronic lymphocytic leukemia, artificial intelligence, ALPODS, flow cytometry

## Abstract

Background: The International Prognostic Index (IPI) is applied to predict the outcome of chronic lymphocytic leukemia (CLL) with five prognostic factors, including genetic analysis. We investigated whether multiparameter flow cytometry (MPFC) data of CLL samples could predict the outcome by methods of explainable artificial intelligence (XAI). Further, XAI should explain the results based on distinctive cell populations in MPFC dot plots. Methods: We analyzed MPFC data from the peripheral blood of 157 patients with CLL. The *ALPODS* XAI algorithm was used to identify cell populations that were predictive of inferior outcomes (death, failure of first-line treatment). The diagnostic ability of each XAI population was evaluated with receiver operating characteristic (ROC) curves. Results: *ALPODS* defined 17 populations with higher ability than the CLL-IPI to classify clinical outcomes (ROC: area under curve (AUC) 0.95 vs. 0.78). The best single classifier was an XAI population consisting of CD4+ T cells (AUC 0.78; 95% CI 0.70–0.86; *p* < 0.0001). Patients with low CD4+ T cells had an inferior outcome. The addition of the CD4+ T-cell population enhanced the predictive ability of the CLL-IPI (AUC 0.83; 95% CI 0.77–0.90; *p* < 0.0001). Conclusions: The *ALPODS* XAI algorithm detected highly predictive cell populations in CLL that may be able to refine conventional prognostic scores such as IPI.

## 1. Introduction

Chronic lymphocytic leukemia (CLL) is the most common leukemic disease in Western countries [1]. The WHO Classification for 2022 categorized CLL as a mature B-cell neoplasm [2]. CLL is diagnosed by the characteristic immunophenotype in multiparameter flow cytometry (MPFC) B-cell panels [3,4]. The prognosis of CLL is heterogeneous; some patients will not require treatment and some patients will progress quickly and some transform into high-grade lymphoma (Richter syndrome). Traditionally, the Rai and Binet classifications of CLL were used for clinical staging and to estimate the prognosis based primarily on leukemia burden [5,6]. In addition to the disease burden, genetic factors such as *TP53* mutation or 17p deletion, 11q deletion, and a complex karyotype indicate a poor prognosis, while deletion of 13q14 and trisomy 12 harbors a favorable prognosis [7,8,9]. Furthermore, mutations in the *NOTCH1*, *SF3B1*, and *BIRC3* genes are associated with shorter survival [10,11,12,13]. Expression of CD38 and ZAP-70 on CLL cells has been associated with unmutated *IGHV* and higher levels of beta2-microglobulin indicating an adverse prognosis [14,15,16,17,18,19,20].

In 2016, the International Prognostic Index for patients with chronic lymphocytic leukemia (CLL-IPI) was introduced [21]. Four prognostic subgroups based on five independent factors (*TP53* status, *IGHV* mutational status, serum beta2-microglobulin concentration, clinical stage, and age) were defined [21]. Five-year overall survival ranges from 93.2% (low-risk CLL-IPI) to 23.3% (very-high-risk CLL-IPI) [21].

However, the five parameters of the CLL-IPI or single markers such as CD38 and ZAP-70 may not reflect the genetic, pathophysiological, and prognostic heterogeneity of the CLL at the individual level compared to structures in large and complex datasets.

The MPFC of peripheral blood from patients with CLL generates individual and complex high-dimensional data from malignant CLL cells and non-malignant surrounding white blood cells. MPCF data from CLL patients are acquired routinely for diagnostic purposes.

Dimensionality reduction techniques such as self-organizing maps (SOM) have already been applied to enhance the interpretation of MPFC data [22,23]. Furthermore, the Citrus (cluster identification, characterization, and regression) algorithm can be applied to find cell-type specific differences between groups in MPFC data [24,25].

Our group described the algorithmic population description approach (ALPODS) which is based on explainable artificial intelligence (XAI) and has been used for classification tasks. The XAI provides sample-based explanations of its decisions by visualizing the immune cell populations which were distinctive for bone marrow compared to peripheral blood [26,27]. ALPODS delivered disjunct cell populations which can be visualized in usual flow cytometry two-dimensional dot plots of FCS data files. This enables human flow cytometry operators to classify these cell populations by conventional MPCF gating.

MPFC data of diagnostic B-cell panels should include information about the individual CLL prognosis and outcome. Therefore, we used the ALPODS algorithm to identify the crucial cell populations that are overrepresented in CLL patients who experienced death or failure of the first line of systemic therapy [26].

## 2. Materials and Methods

### 2.1. Patients, Data Acquisition, and Processing

MPFC data of the peripheral blood from 157 unselected patients with CLL diagnosis were re-analyzed for this study and matched to clinical data. MPFC data were acquired for routine diagnostic analysis at the University Hospital Marburg from 2014 to 2020. The study was approved by the local ethics committee in Marburg. Clinical data included the following: CLL-IPI (i.e., *TP53* mutation status, *IGHV* mutation status, age, Binet, beta2-microglobulin), sex, ECOG, Richter transformation, treatment, date of death, treatment failure, and last follow-up. In case of incomplete or unknown CLL-IPI parameters, half of the score points of the missing parameters were given. The patients were separated into a group with an inferior outcome and a group with a superior outcome. Patients who died during follow-up and had a failure of the first-line systemic therapy were categorized as TTF 1 (time to first-line treatment failure), that is, inferior outcome. All other patients were classified as TTF 0 which indicated a superior CLL outcome.

We used the ALPODS XAI algorithm [26] to identify cell populations in flow cytometry data that were over or underrepresented in patients with the inferior result (TTF 1) or superior outcomes (TTF 0). The predictive value of the XAI populations was compared to the frequency of CD38-positive CLL cells and CLL-IPI on the receiver operating characteristic (ROC) curves. Multiple logistic regression analysis in repeated 10 bootstrap trials was performed for the combination of more than one independent variable to predict dichotomous groups. Therefore, three randomly selected patients from each group (TTF 0 and TTF1) were left out 10 times to test if the results can be generalized for other patients.

### 2.2. Antigen Panel, Flow Cytometry Staining, and Analysis

For diagnosing CLL and other B-cell lymphomas we used a B-cell panel which consisted of two tubes with different fluorescence antibody panels. The first tube (T1) included fluorescence antibodies against B-cell antigens (CD19, CD20, FMC7, CD79b, CD23, light chains of kappa and lambda), T-cell antigens (CD3, CD5, CD2, CD7, CD4, CD8), and the activation marker CD38, which has been described to be prognostic for CLL [14]. The second tube (T2) contained B-cell antigens (CD19, CD20, IgM), markers of hairy-cell leukemia (CD103, CD11c, CD25), follicular lymphoma, and high-grade lymphoma (CD10), and additional markers to ensure the diagnosis of CLL (CD43, CD200). The complete antibody panel, clones, and fluorescence dyes are stated in the Supplementary Information (Table S1).

Two 5 mL polystyrene FACS tubes with fluorescence antibodies in a dried-down layer (DuraClone-Technology, Beckman Coulter, Krefeld, Germany) were incubated for 15 min at room temperature in 100 μL prewashed peripheral blood. After antibody staining, red cells were lysed in 2 mL of VersaLyse™ (Beckman Coulter, Krefeld, Germany) for 10 min, washed with 3 mL of buffered phosphate saline (PBS Biochrom, Berlin, Germany), and centrifuged with 300× *g* for 5 min. The cell pellet was resuspended in 500 μL PBS and measured on a Navios Flow Cytometer (Beckman Coulter, Krefeld, Germany). In total, up to 1 × 10^5^ cells were acquired.

### 2.3. Data Processing

The raw flow cytometry data were compensated, and log transformed. Events with very high side scatter (i.e., mainly granulocytes) were excluded to reduce the amount of data that adds little informative value. Thereafter, data were range standardized between zero and 6 based on the adapted Milligan cooper standardization [28,29]. From the total number of recorded cell events of each sample, a 1% random data set was drawn. Using this 1% sample for training ALPODS a 1000-fold cross-validation was performed. The populations that were relevant for the distinction of TTF 1 versus TTF 0 were selected from ALPODS and the most important populations were filtered using Cohen’s D effect size measure. The computed ABC analysis [30] selected optimal limits for subset division by exploiting the mathematical properties related to the distribution of the items analyzed. ABC analysis divides the data into three disjoint subsets A, B, and C, with subset A comprising very profitable values, i.e., largest data values (“the important few”), subset B comprising values where the yield equals the effort required to obtain it, and the subset C comprising of non-profitable values.

### 2.4. Statistics

The graphs and statistics were compiled with Excel 2016 (Microsoft Corporation, Redmond, WA, USA) and in the R package ggplot2 [31] and DataVisualizations [32], GraphPad Prism^®^ Version 9.4.1 (GraphPad Software, San Diego, CA, USA), R (programming language), www.R-project.org (accessed on 6 October 2022).

## 3. Results

### 3.1. Patient Characteristics

From the 157 CLL patients N = 42 had inferior outcomes (death and/or first-line treatment failure) and N = 115 patients did not reach the defined endpoints (superior outcome, TTF 0). The median age of the total cohort was 68 years (range 26–91 years), and 62 (39.5%) of the patients were female and 95 (60.5%) were male. A total of 83 (52.9%) of the patients were diagnosed with Binet A, 24 (15.3%) with Binet B, and 12 (7.6%) with Binet C. Follow-up was in the median 31.5 months (interquartile range 9–65 months). Additional patient characteristics for the total cohort and separated for TTF 1 or TTF 0 are denoted in Table 1.

### 3.2. Cell Populations Identified by ALPODS

**Figure 1:**
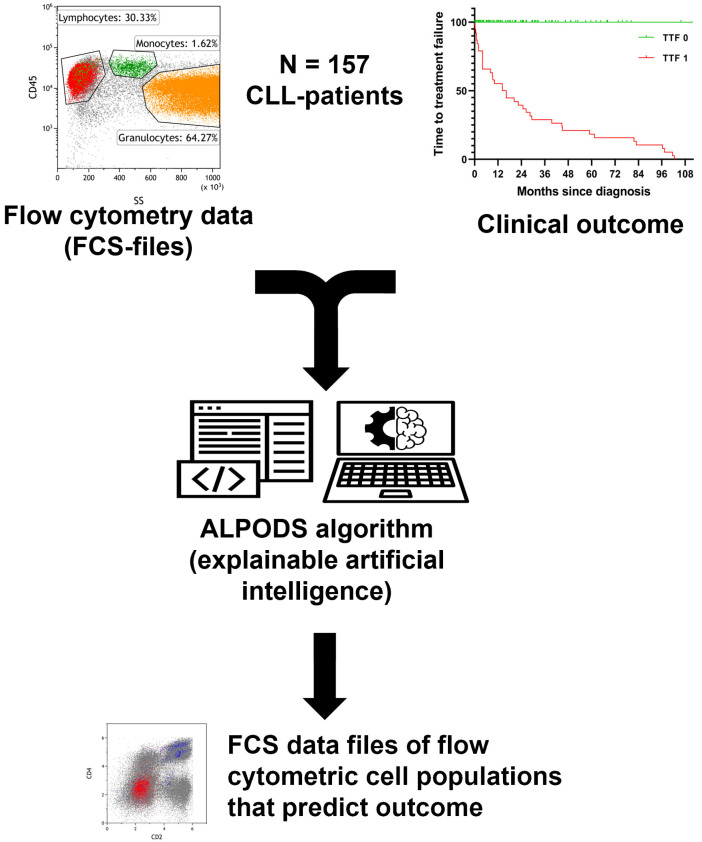
Workflow and data processing. Flow cytometry raw data was standardized and assigned to the outcome group (TTF 1 inferior, TTF 0 superior). The ALPODS algorithm was used to identify distinctive cell populations with different frequencies in TTF 1 versus TTF 0 The most important populations for determination were visualized in flow cytometry bivariate dot plots and assigned to their biological counterparts.

Standardized flow cytometry data and outcome group (TTF 1 or TTF 0) were used as input information for the ALPODS algorithm. ALPODS identified N = 17 distinctive cell populations in the MPFC data which were overrepresented (N = 14/17) or underrepresented (N = 3/17) in the TTF 1 patients’ cohort. Seven out of 17 populations were identified in the first tube (T1) of the diagnostic flow cytometry B-cell panel. Ten out of 17 populations were identified in the second tube (T2) of the B-cell panel. The workflow is depicted in Figure 1.

Mann–Whitney U test and ROC analysis were performed to detect the most predictive XAI populations for inferior outcomes. The results were listed in Table 2. XAI populations with a significant predictive ability for the outcome (TTF 1 vs. TTF 0) were verified for their prognostic value in patients with high IPI (≥4) compared to patients with low IPI (≤1) (Supplementary Information, Table S2). XAI populations with a significant predictive value for the outcome (TTF 1 vs. TTF 0) and the prognosis (IPI low vs. IPI high) were T1C0011, T1C0016, T2C0004, and T2C0018 (bold script in Table 2). Among these populations, solely T1C0016 had a higher frequency in the patients with a good outcome (TTF 0; mean 13.51% vs. 4.91%; SE of difference 1.83) and good prognosis (IPI ≤ 1; mean 12.50% vs. 5.37%; SE of difference 2.42).

In ROC curve analysis, T1C0016 had the highest predictive ability for the outcome of all XAI populations. It should be noted that only the T1C0016 population had the same predictive value as the CLL-IPI score (Table 2; both: AUC 0.78; 95% CI 0.70–0.86). Furthermore, the frequency of CD38-positive CLL cells had a lower predictive ability (AUC 0.66; 95% CI 0.57–0.76, *p* = 0.0018) than all verified XAI populations TC0011 (AUC 0.76; 95% CI 0.68–0.84, *p* < 0.0001), T1C0016 (AUC 0.78; 95% CI 0.70–0.86, *p* < 0.0001), T2C0004 (AUC 0.69; 95% CI 0.58–0.80, *p* = 0.0002), and T2C0018 (AUC 0.73; 95% CI 0.63–0.82, *p* < 0.0001).

**Figure 2:**
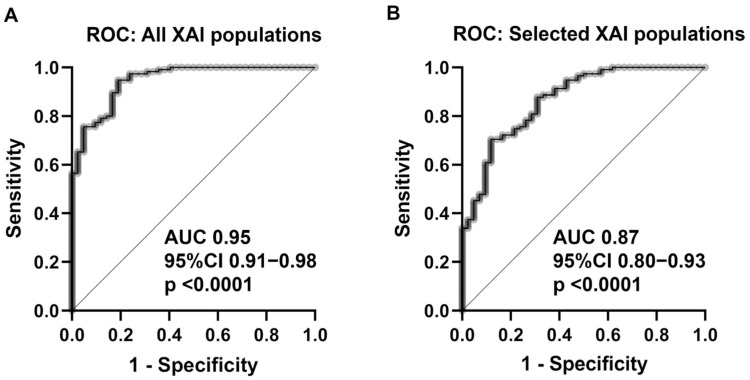
Multiple logistic regression with ROC curve analysis for (**A**) all XAIpopulations revealed a predictive ability of AUC 0.95 (95% CI 0.91–0.98; *p* < 0.0001). (**B**): The restriction of the four most predictive XAI populations for the outcome (TTF) and prognosis (IPI) resulted in lower predictive ability (AUC 0.87; 95% CI 0.80–0.93; *p* < 0.0001). Abbreviations: ROC = receiver operation characteristics; XAI = explainable artificial intelligence; AUC = area under curve; CI = confidence interval.

The 17 XAI populations in combination had a predictive ability of 0.95 AUC (95% CI 0.91–0.98; *p* < 0.0001) for TTF using multiple logistic regression analysis (Figure 2A), which was significantly higher than IPI (*p* = 0.0008; Hanley–McNeil test). Restriction on the four populations of XAI (that is, T1C0011, T1C0016, T2C0004, and T2C0018), which were verified to be predictive of IPI, resulted in a lower diagnostic ability of 0.87 AUC (95% CI 0.80–0.93; *p* < 0.0001) (Figure 2B), but still higher than the conventional IPI (AUC 0.87 vs. 0.78) in this patient cohort, although the difference did not reach statistical significance (*p* = 0.0771; Hanley–McNeil test).

### 3.3. Identification of the XAI-Populations

**Figure 3:**
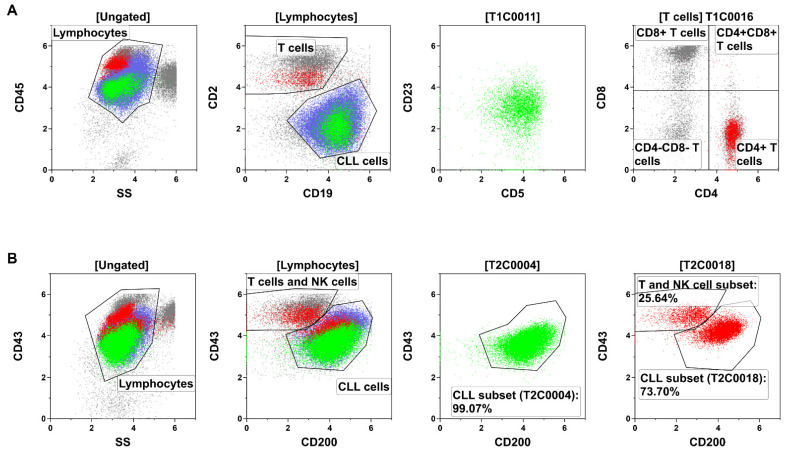
Identification and description of the XAI population through flow cytometry gating in a representative sample. (**A**) The populations T1C0011 (green) and T1C0016 (red) were the most relevant populations for the outcome and prognosis in Tube 1 (T1) of the analyzed B-cell panel. T1C0011 (green) could be located within the CLL cells (blue). The population of T1C0016 (red) corresponded to CD4+ T cells (i.e., T helper cells). (**B**) T2C0004 (green) and T2C0018 (red) were the most relevant populations for the outcome and prognosis in Tube 2 (T2). Both were located mainly within the CLL cells (blue), but T2C0018 was a mixture of a biologically different population (CLL cells and T/NK cells).

The ALPODS algorithm calculated FCS data files that can be depicted with conventional two-dimensional flow cytometry dot plots. Therefore, XAI populations can be gated and analyzed by a human flow cytometry expert. The population T1C0011 has been located within the CLL cells while T1C0016 consisted of CD4+ T cells (Figure 3A). T2C0004 represented nearly exclusively a subset of CLL cells (Figure 3B) and T2C0018 was a mixture of a CLL cell subset (higher fraction) and a T and NK cell subset (lower fraction).

Interestingly, the most relevant cell population for the outcome (T1C0016) in CLL was not part of the malignant cells but consisted of T helper cells, which were overrepresented in patients with a favorable outcome. This observation led to the question of whether increased CD8+ T cells were predictive of an inferior outcome. Indeed, we found that the XAI population T1C0023 consisted of CD8+ T cells (Supplementary Information, Figure S1). T1C0023 was significantly more abundant in patients with an inferior prognosis (IPI ≥ 4 mean 4.30% vs. IPI ≤ 1 mean 0.48; SE of difference 0.77; *p* < 0.00461). However, in the ROC analysis, population T1C0023 was not able to classify between TTF 1 and TTF 0 on its own (AUC 0.53; 95% CI 0.41–0.65; *p* = 0.5573).

### 3.4. Characterization of Predictive Subsets within CLL Cells

**Figure 4:**
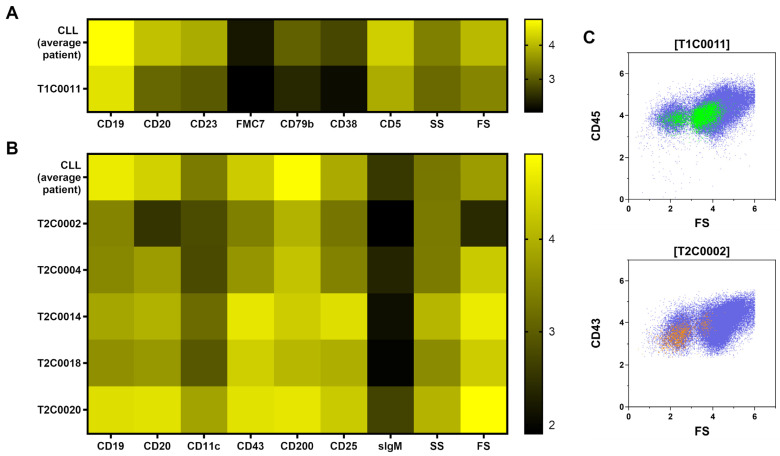
Median antigen expression and scatter properties of CLL subsets with predictive ability for outcome (TTF) were compared with the CLL cells of the average patient. The population T1C0011 (**A**), which was identified in tube 1 (T1), showed lower forward scatter (FS) and diminished antigen expression. T2C0002 (**B**) in tube 2 (T2) shared the lower FS and antigen expression with T1C0011. In flow cytometry dot plots (**C**), parts of T1C0011 and T2C0002 were located in the very low FS region of apoptotic/dead CLL cells. This may indicate a predictive value of apoptotic/dead CLL cells for outcome.

Besides T1C0016 (CD4+ T cells) and T1C0023 (CD8+ T cells), most of the XAI populations were CLL subsets (Tube 1: T1C0011, T1C0012, T1C0017, T1C0019, and T1C0020; Tube 2: T2C0002, T2C0004, T2C0009, T2C0010, T2C0014, T2C0018, and TC0020). The most crucial CLL subsets were T1C0011, T2C0004, and T2C0018, which are shown in Figure 4. Additionally, the median levels of antigen expression and scatter height for relevant subpopulations of CLL cells were compared to the median antigen expression of CLL cells from the average patient in the cohort using a heat map. (Figure 4A,B).

It is noteworthy that the populations T1C0011 and T2C0002 showed a decreased forward scatter expression and a decreased antigen brightness compared to mean CLL cells. Visualized on flow cytometry forward scatter, these populations were located partly in the area of dead and apoptotic cells CLL cells (Figure 4C). This finding suggests that a higher frequency of dead and apoptotic CLL cells is associated with a worse prognosis and outcome. In contrast, the CLL subsets T2C0004, T2C0014, T2C0018, and T2C0020 showed higher antigen expression and scatter light profile than mean CLL cells. In summary, CLL cells with small cell volume (low forward scatter) were overrepresented in MPFC data independent of the B-cell panel tube and indicated a poor outcome. On the contrary, subsets of CLL cells with large cell volumes (high forward scatter) also indicated poor outcomes.

### 3.5. Clinical Significance

The XAI method ALPODS identified 17 cell populations that were effective at predicting outcome. However, correct manual gating of these populations without using ALPODS is sophisticated, especially for the CLL subsets. Exceptions were the populations T1C0016 and T1C0023, which compromised CD4+ T cells and CD8+ T cells, respectively. Both populations were easy to gate manually in flow cytometry dot plots. Therefore, we tested whether T1C0016 (CD4+ T cells) and T1C0023 (CD8+ T cells) add predictive value to IPI and CD38-positive CLL cells. Multiple logistic regression was performed for this four-factor model (Table 3). Odds ratio (OR) >1 favored inferior outcome and <1 favored superior outcome.

In the four-factor model, T1C0023 (OR 1.14; 95% CI 0.95–1.77; *p* = 0.3305) and CD38-positive CLL cells (OR 1.01; 95% CI 1.00–1.02; *p* = 0.1790) were dispensable. IPI and T1C0016 (two-factor model with IPI) showed only slightly lower predictive value than the four-factor model (0.84 AUC; 95% CI 0.77–0.91; *p* < 0.0001 vs. 0.83 AUC; 95% CI 0.77–0.90; *p* < 0.0001). In the two-factor model (IPI and T1C0016) IPI can be replaced by T1C0023 with an acceptable diagnostic ability (0.79 AUC; 95% CI 0.71–0.87; *p* < 0.0001).

## 4. Discussion

In this single-center study, we analyzed immunophenotypes of 157 CLL patients employing an explainable AI (XAI). The XAI identified 17 cell populations in MPFC data which could in combination predict the clinical outcome of CLL with a higher ability than CLL-IPI or the frequency of CD38-positive CLL cells. Most of the 17 cell populations were located completely or in part within the abundant CLL population. However, some cell populations were non-malignant. For example, the T1C0016 population consisted of CD4+ T cells entirely and was underrepresented in patients with a poorer outcome. T1C0016 (CD4+ T cells) was the best single classifier for the outcome of the 17 XAI-identified cell populations. In contrast, T1C0023 compromised CD8+ T cells that were overrepresented in patients with inferior outcomes.

T cells in CLL have been described as dysregulated. CD4+ T cells and CD8+ T cells in patients with CLL deviate from healthy individuals by the accumulation of memory T cells and loss of naïve T cells, increased expression of immune checkpoint receptors (i.e., PD1, TIGIT, CTLA-4), and increased activation [33,34,35,36,37,38]. Inversion of the CD4/CD8 ratio is typical for CLL [39,40,41,42]. Furthermore, Elston et al. showed that patients with a CD4/CD8 ratio >1 have better overall survival and progression-free survival [34]. This is in line with our findings that CD4+ T cells indicated a good outcome and CD8+ T cells an adverse outcome. It would be of interest in further studies to determine which subset of CD4+ cells plays the most significant role in favorable outcomes for patients with CLL. Gating of CD4+ T cells and CD8+ T cells is simple in contrast to XAI populations of CLL subsets and transferrable to other flow cytometry panels that include antibodies against CD4 and CD8. For this reason, we developed a simplified approach to predict the outcome in CLL by the combination of IPI and CD4+ T cells or CD4+ T cells and CD8+ T cells. Both two-factor models discriminate between inferior and superior outcomes in more than 80% of the CLL cases.

In addition to diagnostic ability, the XAI populations provided insight into the immunopathology of CLL. For example, we showed that CLL subsets that predict inferior outcomes were small apoptotic/dead CLL events (T1C0011, T2C0002) with low forward scatter. These results are in line with Witkowska et al. and Jahrsdörfer et al. who described that spontaneous in vitro apoptosis of CLL cells correlated with disease progression and cytogenetics with worse prognosis [43,44].

On other hand, CLL subsets with high forward and side scatter (T2C0004, T2C0014, T2C0018, and T2C0020) were associated with adverse outcomes as well. Forward and side scatter correlates with bigger cell size and internal complexity and suggests the prognostic importance of prolymphocytes in CLL. Oscier et al. described that prolymphocytes >10% in CLL are associated with shorter OS and PFS [45].

There are limitations in this study, as some patients did not have complete data for all components of the IPI. In these cases, a half-point score was assigned, which may result in the inaccurate categorization of some patients. A larger patient cohort is warranted to validate our discoveries in multivariate Cox regression models. This would also allow a better clue, if the results are independent of different treatment strategies. Furthermore, an analysis of other prognostic markers for CLL, such as CD49d and ZAP-70, would strengthen the conclusions of the study [46]. However, in addition to the established markers that have been shown to have prognostic value in CLL, our XAI study provides new insights into the prognostic factors related to the immunology of CLL and the non-malignant, reactive immune system.

## 5. Conclusions

The ALPODS XAI algorithm identified and described highly predictive immune cell populations related to outcomes in CLL. In particular, CD4+ T cells were identified as the best single classifier and improved the predictive ability of CLL-IPI. These findings should be further refined with a different immunophenotyping panel and an independent patient cohort.

## Figures and Tables

**Table 1:** Patient characteristics.

	Total (N = 157)	TTF 1 (N = 42)	TTF 0 (N = 115)
Age (years)			
Median (range)	68 (26–91)	70 (50–88)	67 (26–91)
Sex			
Female	62 (39.5%)	17 (40.5%)	45 (39.1%)
Male	95 (60.5%)	25 (59.5%)	70 (60.9%)
Binet			
A	83 (52.9%)	15 (35.7%)	68 (59.1%)
B	24 (15.3%)	7 (16.7%)	17 (14.8%)
C	12 (7.6%)	8 (19.0%)	4 (3.5%)
unknown	31 (19.7%)	9 (21.4%)	22 (19.1%)
CLL-IPI			
Low	84 (53.5%)	9 (21.4%)	75 (65.2%)
Intermediate	53 (33.8%)	20 (47.6%)	33 (28.7%)
High	16 (10.2%)	11 (26.2%)	5 (4.3%)
Very High	4 (2.5%)	2 (4.8%)	2 (1.7%)
Follow up			
Median (IQR) months	31 (9–65)	71 (38–114)	24 (7–46)
Death (N)	16 (10.2%)	16 (38.1%)	0 (0%)
Treatment failure first line (N)	34 (21.7%)	34 (80.1%)	0 (0%)
Richter’s syndrome (N)	4 (2.5%)	3 (7.1%)	1 (0.9%)
Therapy (first line)			
R-Bendamustin	25 (15.9%)	17 (40.5%)	8 (7.0%)
Ibrutinib	12 (7.6%)	6 (14.3%)	6 (5.2%)
Other	24 (15.3%)	15 (35.7%)	9 (7.8%)
No therapy	80 (51.0%)	0 (0%)	80 (70.0%)
Unknown	16 (10.2%)	4 (9.5%)	12 (10.4%)

Abbreviations: N = number; CLL = chronic lymphocytic leukemia; IPI = International Prognostic Index; TTF = time to first-line treatment failure; IQR = interquartile range; R = rituximab.

**Table 2:** Outcome prediction of the XAI populations.

	TTF 1	TTF 0			ROC		
	Mean (%)	Mean (%)	SE of Difference	*p*-Value (MWU-Test)	AUC	95% CI	*p*-Value
CLL-IPI	2.845 *	1.322 *	0.2712	<0.0001	0.78	0.70–0.86	<0.0001
CD38+	37.05	22.51	5.414	0.0016	0.66	0.57–0.76	0.0018
*XAI-populations*							
Total					0.95	0.91–0.98	<0.0001
**T1C0011**	**5.36**	**1.39**	**0.81**	**<0.0001**	**0.76**	**0.68–0.84**	**<0.0001**
T1C0012	1.91	1.61	0.40	0.2650	0.56	0.45–0.66	0.2635
**T1C0016**	**4.91**	**13.51**	**1.83**	**<0.0001**	**0.78**	**0.70–0.86**	**<0.0001**
T1C0017	6.46	4.15	1.32	0.2136	0.57	0.46–0.67	0.2124
T1C0019	5.19	6.38	1.01	0.0970	0.59	0.47–0.70	0.0966
T1C0020	0.35	0.11	0.08	0.1514	0.58	0.47–0.68	0.1506
T1C0023	2.25	0.57	0.49	0.5593	0.53	0.41–0.65	0.5573
T2C0001	2.59	5.50	1.31	0.0011	0.67	0.57–0.76	0.0012
T2C0002	2.85	0.73	0.61	0.0052	0.64	0.54–0.75	0.0055
**T2C0004**	**9.64**	**1.23**	**1.29**	**0.0002**	**0.69**	**0.58–0.80**	**0.0002**
T2C0009	1.74	0.84	0.49	0.6877	0.52	0.41–0.63	0.6859
T2C0010	0.55	0.21	0.15	0.5369	0.53	0.43–0.64	0.5349
T2C0014	8.19	12.14	1.97	0.0078	0.64	0.54–0.74	0.0081
**T2C0018**	**5.37**	**1.66**	**0.72**	**0.0001**	**0.73**	**0.63–0.82**	**<0.0001**
T2C0020	4.38	1.75	0.63	<0.0020	0.66	0.55–0.77	0.0022
T2C0021	3.25	2.74	0.73	0.4286	0.04	0.44–0.64	0.4266
T2C0028	0.85	0.36	0.14	0.2021	0.57	0.45–0.68	0.2010

* Score points (≠percentage). Abbreviations: TTF = time to first-line treatment failure; ROC = receiver operation characteristics; SE = standard error; MWU = Mann–Whitney U; AUC = area under curve; CI = confidence interval, IPI = International Prognostic Index; T1 = Tube 1 (first tube of the flow cytometry panel; T2 = Tube 2 (second tube of the flow cytometry panel).

**Table 3:** Predictive models for outcome.

	OR	95% CI	*p*-Value	AUC	95% CI	*p*-Value
All XAI populations				0.95	0.91–0.98	<0.0001
CLL-IPI				0.78	0.70–0.86	<0.0001
**Four-factor model**				0.84	0.77–0.91	<0.0001
CLL-IPI	1.53	1.19–2.04	0.0018			
T1C0016 (CD4+ T cells)	0.86	0.78–0.93	0.0003			
T1C0023 (CD8+ T cells)	1.14	0.95–1.77	0.3305			
CD38	1.01	1.00–1.02	0.1790			
**Two-factor model with IPI**				0.83	0.77–0.90	<0.0001
CLL-IPI	1.64	1.29–2.16	0.0001			
T1C0016 (CD4+ T cells)	0.85	0.78–0.92	0.0002			
**Two-factor model w/o IPI**				0.79	0.71–0.87	<0.0001
T1C0016 (CD4+ T cells)	0.85	0.78–0.91	<0.0001			
T1C0023 (CD8+ T cells)	1.29	1.05–2.14	0.1390			

Abbreviations: OR = odds ratio; TTF = time to first-line treatment failure; AUC = area under curve; CI = confidence interval, IPI = International Prognostic Index.

## Data Availability

Not applicable.

## Supplementary Materials

The following supporting information can be downloaded at: https://www.mdpi.com/article/10.3390/curroncol30020148/s1, Table S1: Fluorochrome, antigens, and antibody clones; Table S2: IPI prediction of the XAI populations; Table S3: Multicollinearity analysis of the four-factor model; Figure S1: T1C0023 (CD8+ T cells).; Figure S2: Kaplan–Meyer curve.

## References

[1] Swerdlow S.H., Campo E., Pileri S.A., Harris N.L., Stein H., Siebert R., Advani R., Ghielmini M., Salles G.A., Zelenetz A.D. (2016). The 2016 Revision of the World Health Organization Classification of Lymphoid Neoplasms. Blood.

[2] Alaggio R., Amador C., Anagnostopoulos I., Attygalle A.D., de Oliveira Araujo I.B., Berti E., Bhagat G., Borges A.M., Boyer D., Calaminici M. (2022). The 5th Edition of the World Health Organization Classification of Haematolymphoid Tumours: Lymphoid Neoplasms. Leukemia.

[3] Matutes E., Owusu-Ankomah K., Morilla R., Garcia Marco J., Houlihan A., Que T.H., Catovsky D. (1994). The Immunological Profile of B-Cell Disorders and Proposal of a Scoring System for the Diagnosis of CLL. Leukemia.

[4] Hoffmann J., Rother M., Kaiser U., Thrun M.C., Wilhelm C., Gruen A., Niebergall U., Meissauer U., Neubauer A., Brendel C. (2020). Determination of CD43 and CD200 Surface Expression Improves Accuracy of B-Cell Lymphoma Immunophenotyping. Cytom. B Clin. Cytom..

[5] Rai K.R., Sawitsky A., Cronkite E.P., Chanana A.D., Levy R.N., Pasternack B.S. (1975). Clinical Staging of Chronic Lymphocytic Leukemia. Blood.

[6] Binet J.L., Auquier A., Dighiero G., Chastang C., Piguet H., Goasguen J., Vaugier G., Potron G., Colona P., Oberling F. (1981). A New Prognostic Classification of Chronic Lymphocytic Leukemia Derived from a Multivariate Survival Analysis. Cancer.

[7] Döhner H., Stilgenbauer S., Benner A., Leupolt E., Kröber A., Bullinger L., Döhner K., Bentz M., Lichter P. (2000). Genomic Aberrations and Survival in Chronic Lymphocytic Leukemia. N. Engl. J. Med..

[8] Cramer P., Hallek M. (2011). Prognostic Factors in Chronic Lymphocytic Leukemia—What Do We Need to Know?. Nat. Rev. Clin. Oncol..

[9] Zenz T., Benner A., Döhner H., Stilgenbauer S. (2008). Chronic Lymphocytic Leukemia and Treatment Resistance in Cancer: The Role of the P53 Pathway. Cell Cycle.

[10] Wang L., Lawrence M.S., Wan Y., Stojanov P., Sougnez C., Stevenson K., Werner L., Sivachenko A., DeLuca D.S., Zhang L. (2011). SF3B1 and Other Novel Cancer Genes in Chronic Lymphocytic Leukemia. N. Engl. J. Med..

[11] Rossi D., Rasi S., Fabbri G., Spina V., Fangazio M., Forconi F., Marasca R., Laurenti L., Bruscaggin A., Cerri M. (2012). Mutations of NOTCH1 Are an Independent Predictor of Survival in Chronic Lymphocytic Leukemia. Blood.

[12] Puente X.S., López-Otín C. (2013). The Evolutionary Biography of Chronic Lymphocytic Leukemia. Nat. Genet..

[13] Baliakas P., Hadzidimitriou A., Sutton L.-A., Rossi D., Minga E., Villamor N., Larrayoz M., Kminkova J., Agathangelidis A., Davis Z. (2015). Recurrent Mutations Refine Prognosis in Chronic Lymphocytic Leukemia. Leukemia.

[14] Damle R.N., Wasil T., Fais F., Ghiotto F., Valetto A., Allen S.L., Buchbinder A., Budman D., Dittmar K., Kolitz J. (1999). Ig V Gene Mutation Status and CD38 Expression As Novel Prognostic Indicators in Chronic Lymphocytic Leukemia. Blood.

[15] Hamblin T.J., Orchard J.A., Ibbotson R.E., Davis Z., Thomas P.W., Stevenson F.K., Oscier D.G. (2002). CD38 Expression and Immunoglobulin Variable Region Mutations Are Independent Prognostic Variables in Chronic Lymphocytic Leukemia, but CD38 Expression May Vary during the Course of the Disease. Blood.

[16] Hamblin T.J., Davis Z., Gardiner A., Oscier D.G., Stevenson F.K. (1999). Unmutated Ig VH Genes Are Associated With a More Aggressive Form of Chronic Lymphocytic Leukemia. Blood.

[17] Kröber A., Seiler T., Benner A., Bullinger L., Brückle E., Lichter P., Döhner H., Stilgenbauer S. (2002). VH Mutation Status, CD38 Expression Level, Genomic Aberrations, and Survival in Chronic Lymphocytic Leukemia. Blood.

[18] Rassenti L.Z., Jain S., Keating M.J., Wierda W.G., Grever M.R., Byrd J.C., Kay N.E., Brown J.R., Gribben J.G., Neuberg D.S. (2008). Relative Value of ZAP-70, CD38, and Immunoglobulin Mutation Status in Predicting Aggressive Disease in Chronic Lymphocytic Leukemia. Blood.

[19] Schroers R., Griesinger F., Trümper L., Haase D., Kulle B., Klein-Hitpass L., Sellmann L., Dührsen U., Dürig J. (2005). Combined Analysis of ZAP-70 and CD38 Expression as a Predictor of Disease Progression in B-Cell Chronic Lymphocytic Leukemia. Leukemia.

[20] Heintel D., Schwarzinger I., Chizzali-Bonfadin C., Thalhammer R., Schwarzmeier J., Fritzer-Szekeres M., Weltermann A., Simonitsch I., Lechner K., Jaeger U. (2001). Association of CD38 Antigen Expression with Other Prognostic Parameters in Early Stages of Chronic Lymphocytic Leukemia. Leuk. Lymphoma.

[21] International CLL-IPI Working Group (2016). An International Prognostic Index for Patients with Chronic Lymphocytic Leukaemia (CLL-IPI): A Meta-Analysis of Individual Patient Data. Lancet Oncol..

[22] Van Gassen S., Callebaut B., Van Helden M.J., Lambrecht B.N., Demeester P., Dhaene T., Saeys Y. (2015). FlowSOM: Using Self-Organizing Maps for Visualization and Interpretation of Cytometry Data. Cytom. A.

[23] Saeys Y., Van Gassen S., Lambrecht B.N. (2016). Computational Flow Cytometry: Helping to Make Sense of High-Dimensional Immunology Data. Nat. Rev. Immunol..

[24] Bruggner R.V., Bodenmiller B., Dill D.L., Tibshirani R.J., Nolan G.P. (2014). Automated Identification of Stratifying Signatures in Cellular Subpopulations. Proc. Natl. Acad. Sci. USA.

[25] Polikowsky H.G., Drake K.A., McGuire H.M., Ashhurst T.M. (2019). Supervised Machine Learning with CITRUS for Single Cell Biomarker Discovery. Mass Cytometry: Methods and Protocols.

[26] Ultsch A., Hoffmann J., Röhnert M., Von Bonin M., Oelschlägel U., Brendel C., Thrun M.C. (2021). An Explainable AI System for the Diagnosis of High Dimensional Biomedical Data. arXiv Prepr..

[27] Hoffmann J., Thrun M.C., Röhnert M.A., von Bonin M., Oelschlägel U., Neubauer A., Ultsch A., Brendel C. (2022). Identification of Critical Hemodilution by Artificial Intelligence in Bone Marrow Assessed for MRD Analysis in Acute Myeloid Leukemia: The Cinderella Method. Cytom. Part A J. Int. Soc. Anal. Cytol..

[28] Milligan G.W., Cooper M.C. (1988). A Study of Standardization of Variables in Cluster Analysis. J. Classif..

[29] Thrun M. (2018). Projection-Based Clustering through Self-Organization and Swarm Intelligence: Combining Cluster Analysis with the Visualization of High-Dimensional Data.

[30] Ultsch A., Lötsch J. (2015). Computed ABC Analysis for Rational Selection of Most Informative Variables in Multivariate Data. PLoS ONE.

[31] Wickham H. (2011). Ggplot2. WIREs Comput. Stat..

[32] Thrun M.C., Gehlert T., Ultsch A. (2020). Analyzing the Fine Structure of Distributions. PLoS ONE.

[33] Catakovic K., Gassner F.J., Ratswohl C., Zaborsky N., Rebhandl S., Schubert M., Steiner M., Gutjahr J.C., Pleyer L., Egle A. (2018). TIGIT Expressing CD4+T Cells Represent a Tumor-Supportive T Cell Subset in Chronic Lymphocytic Leukemia. OncoImmunology.

[34] Elston L., Fegan C., Hills R., Hashimdeen S.S., Walsby E., Henley P., Pepper C., Man S. (2020). Increased Frequency of CD4+PD-1+HLA-DR+ T Cells Is Associated with Disease Progression in CLL. Br. J. Haematol..

[35] Palma M., Gentilcore G., Heimersson K., Mozaffari F., Näsman-Glaser B., Young E., Rosenquist R., Hansson L., Österborg A., Mellstedt H. (2017). T Cells in Chronic Lymphocytic Leukemia Display Dysregulated Expression of Immune Checkpoints and Activation Markers. Haematologica.

[36] Tötterman T.H., Carlsson M., Simonsson B., Bengtsson M., Nilsson K. (1989). T-Cell Activation and Subset Patterns Are Altered in B-CLL and Correlate With the Stage of the Disease. Blood.

[37] Peller S., Kaufman S. (1991). Decreased CD45RA T Cells in B-Cell Chronic Lymphatic Leukemia Patients: Correlation With Disease Stage. Blood.

[38] Serrano D., Monteiro J., Allen S.L., Kolitz J., Schulman P., Lichtman S.M., Buchbinder A., Vinciguerra V.P., Chiorazzi N., Gregersen P.K. (1997). Clonal Expansion within the CD4+CD57+ and CD8+CD57+ T Cell Subsets in Chronic Lymphocytic Leukemia. J. Immunol..

[39] Matutes E., Wechsler A., Gomez R., Cherchi M., Catovsky D. (1981). Unusual T-Cell Phenotype in Advanced B-Chronic Lymphocytic Leukaemia. Br. J. Haematol..

[40] Mills K.H.G., Cawley J.C. (1982). Suppressor t Cells in B-Cell Chronic Lymphocytic Leukaemia: Relationship to Clinical Stage. Leuk. Res..

[41] Mittelman A., Denny T., Gebhard D., Cirrincione C., Kurland E., Koziner B. (1984). Analysis of T-Cell Subsets in B-Cell Chronic Lymphocytic Leukemia: A Correlation with the Stage of Disease. Am. J. Hematol..

[42] Platsoucas C.D., Galinski M., Kempin S., Reich L., Clarkson B., Good R.A. (1982). Abnormal T Lymphocyte Subpopulations in Patients with B Cell Chronic Lymphocytic Leukemia: An Analysis by Monoclonal Antibodies. J. Immunol..

[43] Witkowska M., Nowak W., Cebula-Obrzut B., Majchrzak A., Medra A., Robak T., Smolewski P. (2014). Spontaneous in Vitro Apoptosis of de Novo Chronic Lymphocytic Leukemia Cells Correlates with Risk of the Disease Progression. Cytom. B Clin. Cytom..

[44] Jahrsdörfer B., Wooldridge J.E., Blackwell S.E., Taylor C.M., Link B.K., Weiner G.J. (2005). Good Prognosis Cytogenetics in B-Cell Chronic Lymphocytic Leukemia Is Associated in Vitro with Low Susceptibility to Apoptosis and Enhanced Immunogenicity. Leukemia.

[45] Oscier D., Else M., Matutes E., Morilla R., Strefford J.C., Catovsky D. (2016). The Morphology of CLL Revisited: The Clinical Significance of Prolymphocytes and Correlations with Prognostic/Molecular Markers in the LRF CLL4 Trial. Br. J. Haematol..

[46] Bulian P., Shanafelt T.D., Fegan C., Zucchetto A., Cro L., Nückel H., Baldini L., Kurtova A.V., Ferrajoli A., Burger J.A. (2014). CD49d Is the Strongest Flow Cytometry–Based Predictor of Overall Survival in Chronic Lymphocytic Leukemia. J. Clin. Oncol..

