# Supplementary material for: Prediction of Clinical Outcomes with Explainable Artificial Intelligence in Patients with Chronic Lymphocytic Leukemia
Source: Curr Oncol. 2023 Feb 4;30(2):1903–15. doi: 10.3390/curroncol30020148 (PMC9955184; doi:10.3390/curroncol30020148)
Supplement: Supplementary file 1 [file curroncol-30-00148-s001.zip › curroncol-2193677-supplementary.pdf]

Supporting Information:

Table S1. Fluorochrome, antigens, and antibody clones.

Fluorochrome	Tube 1	Tube 2
**FITC**	kappa (RAHK)/ CD8 (B9.11)	CD103 (2G5)
**PE**	lambda (RAHL)/ CD7 (8H8.1)	CD43 (DFT1)
**ECD**	CD23 (9P25)	CD25 (B1.49.9)
**PC5.5**	CD79b (CB3.1) /CD4 (13B8.2)	CD10 (ALB1)
**PC7**	CD5 (BL1a)	CD200 (OX-104)
**APC**	CD38 (LS198-4-3)	
**APC-A700**	CD19 (J3.119)	CD11c (BU15)
**APC-A750**	CD20 (B9E9)/ CD3 (UCHT1)	CD20 (B9E9)
**Pacific blue**	FMC7 (FMC7) /CD2 (39C1.5)	IgM (SA-DA4)
**Krome Orange**	CD45 (J33)	CD19 (J3.119)

Table S2. IPI prediction of the XAI populations.

	**IPI≥4 (high)**	**IPI≤1 (low)**			**ROC**		
	**Mean (%)**	**Mean (%)**	**SE of Difference**	**p-value (MWU test)**	**AUC**	**95%CI**	**p-value**
**CD38+**	**41.35**	**21.36**	**7.55**	**0.0056**	**0.66**	**0.57-0.76**	**0.0018**
eAI-populations							
**T1C0011**	**2.49**	**1.69**	**0.93**	**0.0212**	**0.67**	**0.53-0.80**	**0.0219**
T1C0012	1.69	1.44	0.54	0.5321	0.55	0.40-0.69	0.5281
**T1C0016**	**5.37**	**12.50**	**2.42**	**0.0008**	**0.74**	**0.63-0.84**	**0.0010**
T1C0017	7.03	4.74	1.92	0.4278	0.56	0.41-0.07	0.4237
T1C0019	5.17	6.48	1.52	0.1839	0.60	0.45-0.74	0.1815
**T1C0020**	**0.42**	**0.15**	**0.14**	**0.0080**	**0.69**	**0.55-0.83**	**0.0086**
**T1C0023**	**4.30**	**0.48**	**0.77**	**0.0046**	**0.70**	**0.55-0.86**	**0.0051**
T2C0001	4.01	5.01	1.98	0.9892	0.50	0.36-0.65	0.9868
T2C0002	3.05	0.93	0.97	0.3466	0.57	0.41-0.73	0.3429
**T2C0004**	**8.12**	**1.78**	**1.84**	**0.0029**	**0.71**	**0.58-0.85**	**0.0033**
T2C0009	0.57	1.17	0.42	0.1602	0.60	0.47-0.73	0.1584
T2C0010	0.28	0.32	0.19	0.9169	0.51	0.36-0.66	0.9146
T2C0014	11.27	10.34	2.71	0.8283	0.52	0.37-0.66	0.8238
**T2C0018**	**4.71**	**2.27**	**0.99**	**0.0030**	**0.71**	**0.60-0.82**	**0.0034**
T2C0020	4.34	2.00	0.89	0.2346	0.59	0.44-0.73	0.2317
T2C0021	3.16	3.20	1.06	0.9586	0.50	0.36-0.65	0.9572
T2C0028	1.24	0.42	0.22	0.4348	0.56	0.39-0.72	0.4309

Table S3. Multicollinearity analysis of the four-factor model.

Multicollinearity	Variable	VIF	R2 with other variables
$\beta0$	Intercept		
$\beta1$	IPI	1.212	0.1746
$\beta2$	T1C0016	1.039	0.03745
$\beta3$	T1C0023	1.154	0.1333
$\beta4$	CD38	1.054	0.05092

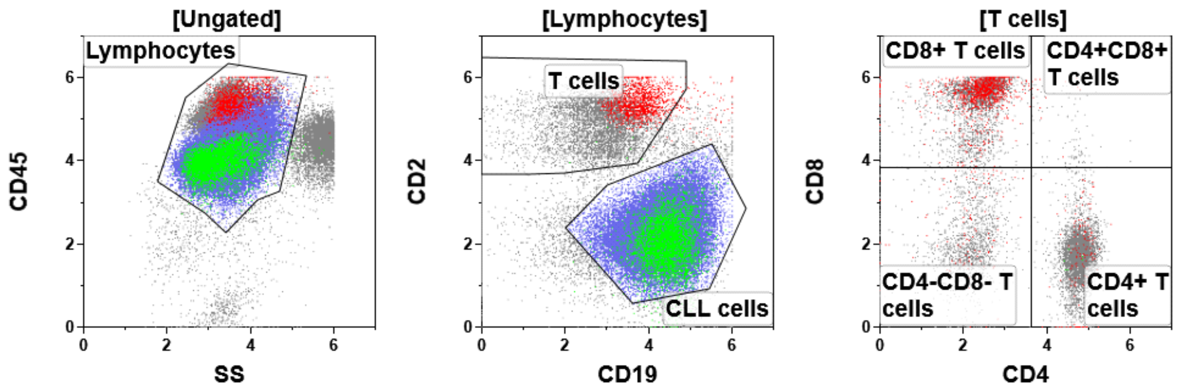

Figure S1. T1C0023 (CD8+ T cells).

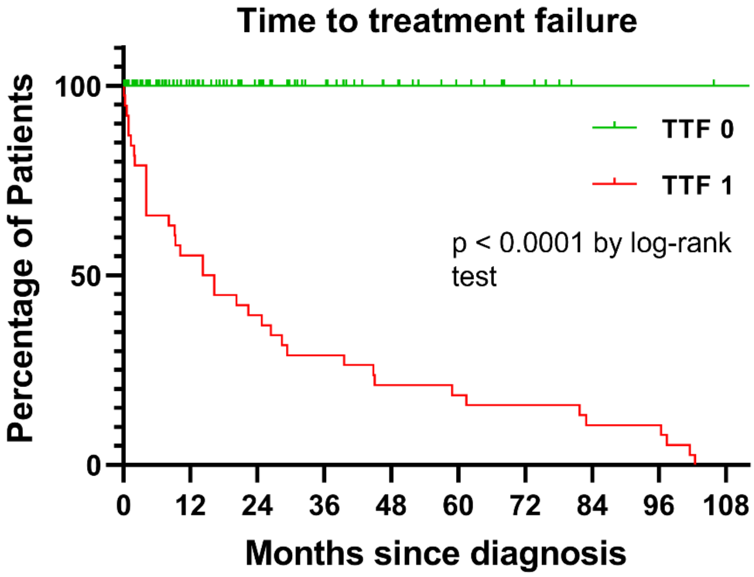

Figure S2. Kaplan-Meyer curve .
